# Transforming the workplace environment to prevent non-communicable chronic diseases: participatory action research in a South African power plant

**DOI:** 10.1080/16549716.2018.1544336

**Published:** 2018-11-26

**Authors:** Darcelle Schouw, Robert Mash, Tracy Kolbe-Alexander

**Affiliations:** a Division of Family Medicine and Primary Care, Faculty of Medicine and Health Sciences, Stellenbosch University, Cape Town, South Africa; b School of Health and Wellbeing, University of Southern Queensland, Ipswich, Australia

**Keywords:** non-communicable diseases, workplace environment, transformation, participatory action research, diversity, leadership

## Abstract

**Background**: The workplace is an important setting for the prevention of non-communicable diseases (NCDs). Policies for transformation of the workplace environment for occupational health and safety in South Africa have focused more on what to do and less on how to do it. There are no guidelines and little evidence on workplace-based interventions for NCDs.

**Objective**: The aim of this study was to learn how to transform the workplace environment in order to prevent and control cardio-metabolic risk factors for NCDs amongst the workforce at a commercial power plant in Cape Town, South Africa.

**Methods**: The study design utilized participatory action research in the format of a cooperative inquiry group (CIG). The researcher and participants engaged in a cyclical process of planning, action, observation and reflection over a two-year period. The group used outcome mapping to define the vision, mission, boundary partners, outcomes and strategies required. At the end of the inquiry the CIG reached a consensus on their key learning.

**Results**: Substantial change was observed in the boundary partners: catering services (78% of progress markers achieved), sport and physical activities (75%), health and wellness services (66%) and managerial support (65%). Highlights from a 10-point consensus on key learning included the need for: authentic leadership; diverse composition and functioning of the CIG; value of outcome mapping; importance of managerial engagement in personal and organizational change; and making healthy lifestyle an easy choice.

**Conclusion**: Transformation included a multifaceted approach and an engagement with the organization as a living system. Future studies will evaluate changes in the risk profile of the workforce, as well as the costs and consequences for the organization.

## Background

Non-communicable diseases (NCDs), are the leading cause of death globally, accounting for 71% of deaths and 85% of premature deaths (< 70 years) in low- and middle-income countries (LMIC) [1–3]. The NCDs include cardiovascular disease, cancer, diabetes and chronic obstructive pulmonary disease, which poses a substantial public health challenge in South Africa [4]. NCDs are responsible for 43% of deaths per year in South Africa, and this is particularly crucial for employers as most of the deaths occur before the age of 65 years [3,5–8]. Since the determinants for NCDs include behavioural, environmental and socio-economic factors, a shift towards a ‘whole of government’ and a ‘whole of society’ approach is needed [9,10].

The workplace has been identified by numerous health authorities as strategic in reaching working adults, as a substantial proportion of the population can be reached from a wide variety of socio-economic and cultural backgrounds. The workplace influences employees’ health, safety and risk behaviour, and can act as an accelerator or preventer of chronic diseases [11]. The workplace affects individual health behaviours through both physical and psycho-social mechanisms [12]. According to the World Health Organization (WHO), a healthy workplace is one where employees and managers collaborate to continually improve the health, safety and well-being of all employees, and by doing this also sustains the productivity of the business [13,14].

There is a growing body of evidence on the success of using health education programmes by health professionals in the workplace [15]. Most of these programmes, however, exclude the environmental changes that help to make healthy choices easy and sustainable [16]. Work environments in South Africa are governed by legislation and policies to protect employees from occupational health hazards [17]. However, these policies have focused more on ‘what’ to do and less on ‘how’ to do [18]. Implementation of these policies in organizations is typically the responsibility of the health and wellness departments [19], which are often embedded in human resource management units within the organizational structure. Leaders of occupational health and safety are unlikely to receive training in the principles of behaviour and environmental change [19]. Stronger leadership and processes that reach beyond the confines of health and wellness services may be needed [20].

Innovative actions in the workplace are needed to tackle the predicted increase of NCDs over the next two decades in South Africa [18]. While there are multiple workplace wellness programmes, there is no South African guideline on preventing NCDs in the workplace and there may be a need for such a guideline to be informed by local evidence. Single-component interventions have minimal impact on the risk of NCDs [21]. Focused attention should be given to multi-component interventions that make healthy eating and physical activity part of employees’ daily schedules [22–25] Programmes also succeed where employees are involved in the planning and implementation [11,26]. There is therefore a need to be innovative in designing, implementing and evaluating interventions to prevent NCDs in the workplace. The need for such an intervention was also evident in the annual health risk assessments performed by the commercial power plant that is the focus of this study.

This study aimed to explore how to transform the workplace environment at a commercial power plant in South Africa in order to prevent and manage the risk factors for NCDs amongst the workforce.

## Methods

### Study design

Participatory action research (PAR) [27] in the format of a cooperative inquiry group (CIG) followed a cyclical process of planning, action, observation and reflection over a two-year period (November 2015–December 2017).

### The setting

This study was conducted at a commercial industrial power plant in the Western Cape, South Africa. The industry’s focus was technical, with 1,743 employees that included engineers, plant operators, physicists, technicians, artisans and support staff. The organization was governed by strict policies and standardized operating procedures, although it also experienced severe budget constraints, and was headed by a general manager with an executive committee.

Health in the South African workplace is governed by the National Occupational Health and Safety Act of 2003 [28]. The plant included a health and wellness department whose mandate was to provide occupational health services and medical surveillance, assess fitness to work and promote wellness. The staff included a medical practitioner, seven occupational health nurses, a wellness manager, a senior adviser for physical wellness (the first author) and two administrators.

The plant was a critical part of the national electricity supply, but also had to close down generation on a regular basis for maintenance. During these ‘outages’, employees had to work shifts and extended hours in order to minimize the duration of the outage, which also impacted their health and wellness.

All employees received subsidized meals from an external caterer contracted to the organization. The power plant was located within a nature reserve, but there were no recreational or physical activity facilities at the workplace. A previous survey of employees’ health risks found that they had multiple risk factors for NCDs and underlying risky behaviours such as harmful or dependent alcohol use (29%), tobacco smoking (26%), inadequate fruit and vegetable intake (73%) and physical inactivity (64%) [29]. These four risk factors became the target for the design of the intervention.

### Forming the co-operative inquiry group

Eleven employees in managerial positions were purposefully invited to join the CIG based on their influence over decision making, as well as their track record of successful action and openness to change. Of those invited, two declined as they felt overcommitted. Participants attended an onsite presentation on NCDs, the purpose of the inquiry and the process of the CIG. All participants consented to take part for two years. The CIG was facilitated by Darcelle Schouw (DS) and co-facilitated by Bob Mash (BM). CIG members were comprised of one financial manager, one wellness manager, one senior occupational health nurse, three engineering managers, two project management advisors, one industrial relations manager and one human resources manager, one quality control officer, and a manager from the organization’s training department.

### Initiating the inquiry

The CIG aligned itself with the main research question, ‘How can the workplace environment be transformed in order to prevent and control the risk factors for NCDs amongst the workforce?’. Ownership of the inquiry by the whole group, as well as democratic and collaborative group dynamics, was encouraged (see Table 4, Supplementary file on quality criteria for the functioning of the CIG). Group members were trained in reflectivity as they were both the researchers and the researched during this process and needed to document their observations and reflections. At the same time, group members were encouraged to engage with practical action and transformation of practice at the plant.

**Figure 1.**
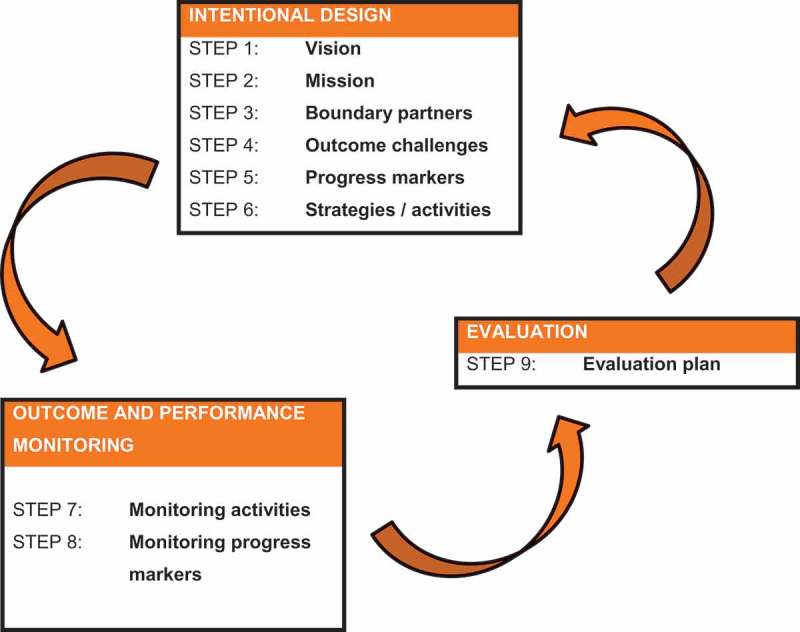
Phases and steps of outcome mapping. Source: Earl et al. 2001[30].

The CIG used outcome mapping (OM) to identify the intended outcomes, plan initial activities and subsequently monitor progress during the inquiry [30]. OM involved three phases: the intentional design, outcome and performance monitoring, and evaluation (Figure 1). The intentional design phase answered four questions: What is the vision and mission of the project? Who are the boundary partners? What are the changes (the outcomes) being sought? How will the project enable these changes (the activities)? This design also provided a framework for the monitoring of outcomes and activities. During the evaluation phase at the end of the CIG, a consensus was built regarding the key learning from CIG members over the two years.

Boundary partners were people or groups that the CIG needed to influence in order to achieve its mission. The four boundary partners were:

- The contracted caterer and food supplier
- External support services such as the surrounding nature reserve, sport clubs and health services
- The health and wellness department
- The management and decision makers

The CIG agreed on outcome challenges for each boundary partner, which described the desired changes in behaviour. Each outcome challenge was then broken down into a series of progress markers, which reflected increasing complexity and depth of change and were categorized into what the CIG would like to see, expect to see and love to see [27,30]. The full intentional design using OM is given in a supplementary file.

**Table 1.** Consensus on key learning from CIG members on transforming the workplace environment.

Key learning of the CIG	Score
Dynamic, passionate leadership of CIG; collaborative, focused, supportive.	35
Composition, network and diversity of CIG; multiple foci/sub-groups (not necessarily in expert field but interest – creativity, access points to organization).	24
Having a clear set of outcomes/goals in outcome mapping.	24
Creating tangible opportunities to take action, and an environment that stimulates participation by the workforce.	17
Finding a way to personally engage and motivate management	13
Functioning of CIG is different to other groups. CIG is organic, creative; more personal engagement, fewer rules/procedures.	8
CIG takes positive, energetic, innovative approach ‘out of the box’. Making change with minimal resources.	8
Included key people linked to the boundary partners as partners in the process/activities.	8
Good functioning of CIG. Participation, integration, execution.	7
Having a wellness champion in each department who wants to see the change, not necessarily management.	6
Having a specific separate group of people committed to the issue.	6
Having a health risk profile assessment at baseline for individuals to change focus on what to do.	4
People selected had sufficient autonomy, dedication, capability and confidence regarding their work to participate in CIG.	3
People are valued in CIG, not just the task.	2
Problem solving between boundary partner groups was focused, yet took responsibility for ‘bigger-vision’ picture. Took ownership of the group.	2

CIG = Cooperative inquiry group. Ranked according to the score derived from the nominal group technique.

#### Action and observation

After the initial planning using OM, the CIG engaged with the activities that were identified for each boundary partner. The principal researcher also shared relevant evidence from the literature on activities that had worked elsewhere. The CIG divided itself into a number of smaller sub-groups who each focused on a different boundary partner and met more often than the CIG as a whole. The whole CIG met monthly for two to four hours at the workplace over this period, equating to 22 meetings.

#### Reflection and planning

Each CIG meeting started with a report back from members on what they had done and observed. The CIG then reflected on what had happened in order to learn how to transform the organization and to inform planning for further action. A set of structured questions was used to guide reflection. These included: What did I do? What happened? What was different to what I expected? What did I not do? What did I do instead? What have I learnt from this? What action can I take in future? The meeting ended with planning new or adjusting existing actions for implementation in the sub-groups.

### Documentation of research

Each member of the CIG kept a journal of their observations and reflections. All the CIG meetings were documented by means of audio recordings, materials from small-group work, such as key points on newsprint or drawings, and notes taken during the meetings by the facilitator. These various data sources were used to summarize the observations, reflections and plans from each meeting. The researcher made a summary of each meeting and distributed it to the team for validation.

#### Building of final consensus

The CIG concluded by building a final consensus of what was learnt over the two years. A member of the CIG delivered a presentation on what had happened with each boundary partner in relation to the progress markers and outcome challenge. The whole group then reflected on the extent of change and individually scored each progress marker from 0 (no change) to 3 (fully achieved). The amount of change achieved was evaluated as a percentage of the total possible score that could be achieved for that boundary partner if all members of the CIG scored every progress marker as fully achieved. The CIG then reviewed the strategies that had been attempted to achieve these changes and reflected on which strategies had worked and which had not. Finally, the CIG engaged with a nominal group technique to identify the key lessons learnt and to rank them from most to least important [31,32]. In the nominal group technique each person scored their top five lessons from most important (score = 5) to least important (score = 1). The sum of the scores from all participants was then used to rank the items. The summary of the final consensus meeting was validated by the whole group.

## Results

**Figure 2.**
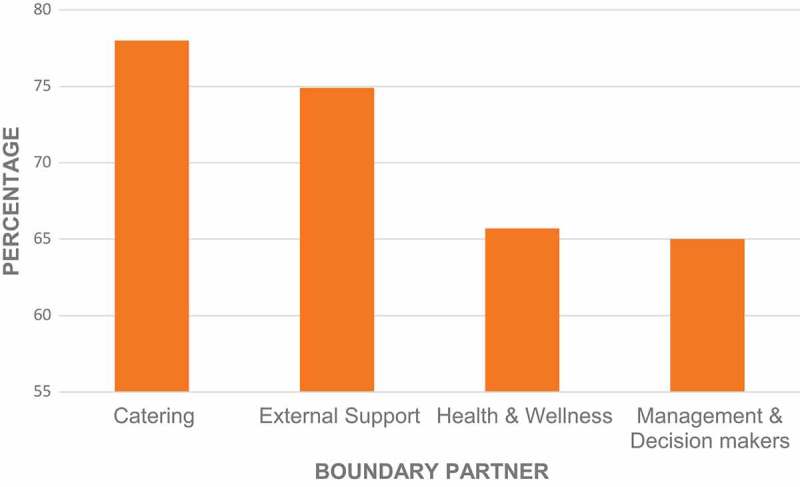
Percentage of change in each boundary partner. Note: Percentage of all progress markers achieved for each boundary partner. Each progress marker was scored 0 (no change), 2 (partially achieved) or 3 (fully achieved). Percentage is calculated as the score/total possible score x 100.

The scoring of the progress markers in the four boundary partners is indicated in Figure 2. Change was seen in all boundary partners, although scores were highest in catering and external support and lowest in health and wellness, as well as management.

The following sections describe the changes seen in each boundary partner and the activities that enabled these changes. Illustrative quotations from the CIG that support the key findings are given in the supplementary file.

### Caterer and food supplier

The CIG informed their planning and actions by benchmarking with five similar industries to understand how they were incorporating healthy meals into their catering. Strategic meetings were held between the finance manager (a member of the CIG), contract manager and external catering manager. Despite initial resistance the team was able to negotiate the inclusion of a ‘wellness’ meal at no additional cost.

The new wellness meal was planned with the dietician and the catering company provided colourful posters on healthy food choices and the wellness meal. The wellness meal was positioned at the top of the computer touch screen as the first option to consider, with healthy foods coded in green and gold. The launch of the wellness meal was celebrated with a Valentine’s Day picnic for all employees, with ‘healthy heart’ messages. Within three months the wellness meal, which was initially offered once a week, was offered every day.

The catering staff were trained in the preparation of wellness meals and consulted on all changes to ensure collaboration and buy-in. When the wellness meal became somewhat monotonous the chef prepared a ‘taste off’ for employees to give feedback on a variety of other meal options.

Live demonstrations on how to prepare healthy food at home were broadcast on the plasma screens to all employees at their workstations. The health and wellness department also promoted healthy eating and provided health education to employees, with relevant materials [33].

### External service providers

The CIG members presented a strategy for promoting physical wellness in the employees to the general manager, who subsequently acted as sponsor for the implementation of the program.

Areas were identified within the surrounding nature reserve for walking, running and cycling. Management provided support for extended lunches on Wednesdays to encourage employees to participate in physical activity and a monthly Friday sport day. The ‘First Friday’ sports day, with innovative themes, was incorporated into the organization’s official calendar and staff received time off from work to participate. Private health insurers provided medals for the monthly sports day and marketed their own wellness programmes to employees. Functional exercise equipment was purchased for the staff to utilize during their lunchtimes under supervision. The CIG also linked employees and their families with weekend park runs organized in local communities. A weight loss challenge, based on the television series *The Biggest Loser*, was launched throughout the organization, with 18 interdepartmental teams participating. The CIG group began the process for a new gym facility; however, this was not implemented during the study period.

The interventions resulted in team building and improved morale, as reported by employees and management. A significant shift, according to reflections of the CIG, was evident in the volume of employees exercising before and after work, walking during their lunchtimes, and ordering from the wellness menu.

The communications department promoted physical activities and sport events using innovative signage and marketing. The CIG also collaborated with the Western Cape government’s Working on Wellness programme for employees to access activities such as Pilates, spinning, yoga and kickboxing outside of the work environment. All employees, who were screened for health risks received weekly text messages with health tips and motivation for behaviour change throughout the study period.

### Health and wellness

The CIG planned activities in collaboration with health and wellness staff, local hospitals, clinics, and private health insurers. Initially, the support from the health and wellness department was minimal as they were uncomfortable with the CIG implementing strategies to prevent NCDs, because they perceived this as their role.

In order to effectively educate employees on the benefits/importance of healthy eating and physical activity in promoting a healthy lifestyle, CIG members collaborated with the Information Technology and Communication Departments to educate employees on healthy living. A dedicated newsletter was distributed to all employees on a quarterly basis; this focused on healthy eating and physical activity. In addition, private health insurers conducted health risk assessments and provided feedback to employees, specifically those identified at high risk for NCDs to identify employees at high risk, and referred them to the health and wellness department. Clinical staff participated in a three-day training course on brief behaviour change counselling to improve their capability to support individual lifestyle change.

Visibility and greater uptake of the health services was achieved by taking the annual health risk assessment to the employees and providing them with feedback and counselling at their workstations, rather than expecting them to come to the department.

### Managers and decision makers

In 2016, three months after the CIG was formed, two presentations were made at executive level to inform management on the organizational impact of NCDs and how transformation of the environment could improve productivity and well-being of staff. The meeting was experiential because managers were also medically assessed and informed of their personal health risk profiles. Managers approved all strategies presented, although the CIG had to be creative in planning low-cost initiatives as there was no substantial budget allocation.

Healthy lifestyle was promoted in meetings and work team sessions. Managers led by example in choosing wellness meals, marketing activities, participating in physical activities and promoting health by broadcasting discussions of their own behaviour change on plasma screens throughout the plant. Health and wellness also became a permanent agenda item for the monthly employee meetings hosted by the general manager.

The capacity-building programme for supervisors was also revised to include a wellness segment that featured personal risk assessment, education on healthy eating, physical activity and coping with stress. The physical activities were also included in the business plan as they were seen to uplift staff morale and team building.

A ‘winning outage well’ campaign was initiated to improve employees’ health during scheduled maintenance of the plant, which occurred twice during the study period for three months each time. The CIG was included in the outage planning meetings to monitor and give feedback on employees’ wellbeing and make recommendations. Healthy food was provided weekly during the outages to employees by senior management promoting health in sporting attire.

### Consensus of CIG

Consensus of key lessons learnt by the CIG are shown in Table 1. The key lessons learnt emphasized the importance of the CIG itself in enabling change. The CIG was unlike other project groups in that it was highly diverse in terms of its members’ educational, departmental and cultural backgrounds. Change was enabled by harnessing this diverse set of ideas, competencies and circles of influence, which went far beyond the health and wellness department. The group dynamics were also highlighted in terms of the lack of hierarchy, mutual respect, collaboration, open communication, alignment with purpose and shared values. The leadership of the group was also critical in connecting, motivating and encouraging creativity and innovation. Members of the CIG took ownership of the project and felt passionate about facilitating change. The CIG also enabled the informal identification of wellness champions in each department.

The OM process enabled the CIG to have clear goals and strategies, and to conceptualize the whole project as a group, particularly at the beginning. Subsequently, the OM enabled the CIG to check on progress and to revise goals or strategies.

Management buy-in enabled change to happen with all the other boundary partners. It was important for them to engage at both a personal and organizational level. Creating practical, fun and easily accessible activities or healthy lifestyle options was also critical to enabling change.

## Discussion

Transformation of the workplace environment was achieved using a PAR approach. The fundamental enablers were functioning of the CIG; value of outcome mapping; management support; and making the healthy choice the easy choice. The intervention as a whole was subsequently named the ‘Healthy Choices at Work’ programme by the organization.

Transformation was enabled by team diversity and authentic leadership [34]. The team was diverse in terms of age, gender, professional and cultural background, as well as position within the organization. Health programmes that are driven by a diverse set of experts rather than only health professionals may be more effective at solving complex problems because they engage multiple disciplines [35,36]. The CIG, therefore, worked in a transdisciplinary approach, which is defined ‘as research efforts conducted by investigators from different disciplines working jointly to create new conceptual, theoretical, methodological, and translational innovations that integrate and move beyond discipline-specific approaches to address a common problem’ [37]. The CIG can be seen as exemplifying a transdisciplinary approach as it integrated people from health, physical therapy, engineering, finances and human resource management into one team that addressed the research question collectively and collaboratively [37].

The CIG identified a style of leadership that was congruent with the theory of authentic leadership [36]. This could be understood in terms of an emphasis on enhancing trust, collaboration and acceptance of others’ differences, as well as meaningful recognition, effective communication and appreciation of others’ contributions [19,24,34]. In addition, authentic leadership enhances commitment and engagement of group members, which leads to better performance and achievement of outcomes [35,36,38]. The functioning of the CIG was congruent with John Maxwell’s 4Cs of effective teamwork: the importance of group chemistry, character that makes trust possible, belief in the capacity to make a difference, and members contributing beyond their job responsibilities [39].

The outcome mapping approach was particularly congruent with the CIG because it defined outcomes as ‘changes in the behaviour, relationships, activities, or actions of the people, groups, and organizations with whom a program works directly’, rather than changes in biological or health indicators [30]. OM focuses on measuring the contribution of programmes to complex outcomes, rather than trying to attribute change to specific interventions. OM therefore was well aligned with planning transformation in a complex organizational environment with a focus on changing risk behaviour.

Management endorsed the initiative at the highest level, which enabled change to occur at all other levels in the organization. Managerial support was also important because it went beyond this traditional approach to change through the setting of policy or issuing of directives. The modelling of personal commitment to behaviour change and visible demonstration of managerial support was important [40].

Many of the strategies related to healthy eating and physical activity involved making the healthy choice an easy choice. Social economists have described how changing the choice architecture or environmental design, rather than a person’s motivation, can ‘nudge’ people in the desired direction. Examples of small changes to the choice architecture at the power plant included placing the wellness meal at the top of the computer screen, giving permission for employees to engage with physical activity during working hours and providing them with a range of options in the immediate vicinity [41].

Current thinking on organizational change highlights the need to see the organization as a complex adaptive or living system, as well as in terms of the more traditional hierarchical and mechanistic structure [42]. The successful approach to transformation in this study was more congruent with the former viewpoint as it emphasized networking and relationships, and feedback loops, and allowed change to emerge from the creation of a non-hierarchical community of practice. For transformation to take place leaders must become less hierarchical and shift their thinking from control to connectivity [42].

The behaviour change wheel is a conceptual framework that helps to make sense of the interventions used by the CIG [43]. The framework provides a typology of different types of interventions and policy changes that can support behaviour change. In this study, the activities developed by the CIG can be allocated across all the different types of interventions and policy categories, as shown in Table 2 [43]. This speaks to a multifaceted approach and resonates with evidence that organizations will benefit more from multi-component programmes to change behaviour [40,44].

**Table 2.** Definitions of interventions and policies.

Intervention	Definition	Examples
Education	Increasing knowledge or understanding	Information was provided to staff to promote healthy living
Persuasion	Using communication to induce positive or negative feelings or stimulate action	Live demonstrations of exercise and healthy eating were provided on plasma screens broadcasted to all employees. Live interviews with managers were aired on screens about their healthy lifestyles.
Incentivization	Creating expectation of reward	Using prizes for ‘biggest loser’ competitions, and monthly walks, runs and cycling in the nature reserve to improve physical fitness and reduce weight
Training	Imparting skills	Brief behaviour-change training done with all health and wellness staff
Restriction	Using rules to reduce the opportunity to engage in the target behaviour (or to increase the target behaviour by reducing the opportunity to engage in competing behaviours)	Prohibiting the use of salt in the preparation of food
Environmental restructuring	Changing the physical or social context	Employees received three NCD related text messages per week on their cellphones to assist in making healthy choices.
Modelling	Providing an example for people to aspire to or imitate	Management and CIG model healthy lifestyles by participating in and leading the planned activities
Enablement	Increasing means/reducing barriers to increase capability or opportunity	Behavioural support to reduce NCDs by the health and wellness team; regular exercise classes, weight loss and fitness challenges
Policies		
Communication/marketing	Using print, electronic, telephonic or broadcast media	Quarterly wellness newsletter, onsite billboard advertising, emails, plasma screens, meetings
Guidelines	Creating documents that recommend or mandate practice. This includes all changes to service provision.	Produce directive for catering wellness meals. No meals and snacks to be issued to staff without wellness approval.
Regulation	Establishing rules or principles of behaviour or practice	Time off is given once a month for all employees to participate in three-hour sports sessions
Environmental/social planning	Designing and/or controlling the physical or social environment	The gym was planned using project managers, design and structural engineers
Service provision	Delivering a service	Support services were established by the medical aids, health and wellness team, sport and recreation, and catering department

### Strengths and limitations

Reflecting on the eight quality criteria for cooperative inquiry [27], the CIG members were well aligned with the purpose of the inquiry and took ownership of the process. The facilitator ensured a collaborative and democratic group process by valuing each person’s contribution, openness and being truthful without judgement. The CIG members were more committed to practical action than in-depth reflection and needed assistance to document their actions and structure reflection. The groups’ findings are clearly described so that other organizations can transfer ideas and learning to their own contexts. New knowledge was created from the experience of the CIG members during the inquiry, and more particularly through a formal consensus-building process. Although CIG members were employees, their reflections and findings were not influenced by the organization and were not part of the appraisal of their key performance areas. The nature of PAR is that participants are engaged in changing their own practice, and therefore it was essential that CIG members were integral to the environment being transformed.

The learning from this study may be replicable in other business settings, particularly large enterprises that have the ability to engage with all the activities. Nevertheless, many of the activities would be possible for medium-sized enterprises, such as the provision of healthy food, opportunities for physical activity and engagement with management.

### Recommendations

The study findings point to the following recommendations:

1. Create a diverse, multi-professional, transdisciplinary team to lead the transformation process.
2. Lead in a style congruent with authentic leadership that encourages collaborative teamwork.
3. Ensure that management is actively involved in both a personal and a professional capacity.
4. Plan and monitor transformation with tools that are consistent with the behavioural goals and complexity of change; for example, outcome mapping.
5. Take a systematic approach to change that works through communities of practice, as well as traditional hierarchies.
6. Design multifaceted interventions and policy changes.
7. Design interventions that make the healthy choice the easy choice within the organizational environment.

The intervention focused primarily on cardio-metabolic risk factors for NCDs, and in the future it may be important to focus more directly on mental health, particularly as the industry considered here is working under considerable political, economic and psycho-social pressures.

- These findings may be useful to the development of policies or guidelines by the Department of Health on the prevention of NCDs in the workplace in South Africa.
- Transformation is an ongoing process and the CIG has continued to work together even though the formal research phase has been completed.

Further research will be published on evaluating change in risk factors for NCDs within the workforce, and quantifying the incremental costs and other organizational consequences of the intervention.

## Conclusion

Transformation of the workplace environment was documented through changes in catering, opportunities for physical activity, management, as well as the health and wellness department. Key factors in enabling transformation were the functioning of the CIG, value of outcome mapping, personal and professional managerial support, as well as making the healthy choice the easy choice in the organizational environment. Planning multifaceted interventions, as well as engaging the organization as both a living system and a hierarchy, were thought to be important. Further research should measure actual change in risk behaviour, risk factors, and the costs and consequences for the organization.

## Paper context

Transformation of the workplace to prevent non-communicable diseases is usually associated with single-component interventions by trained health professionals. This paper reports that through a participatory process incorporating diversity, workplace transformation can be achieved using multi-component interventions. In addition, transformation was made possible using limited resources and innovative strategies.
