# Supplementary material for: Transforming the workplace environment to prevent non-communicable chronic diseases: participatory action research in a South African power plant
Source: Glob Health Action. 2018 Nov 26;11(1):1544336. doi: 10.1080/16549716.2018.1544336 (PMC6263095; doi:10.1080/16549716.2018.1544336)
Supplement: Supplemental Material [file ZGHA_A_1544336_SM8793.docx]

**ZGHA – 2018-0217**

**Outcome mapping: intentional design**

**Introduction**

Outcome mapping (OM) supported the cooperative inquiry group (CIG) process through its three phases: intentional design, monitoring the outcomes and performance, and evaluating what was learnt. This supplementary document describes the intentional design phase.

The CIG used outcome mapping to define the vision, mission, boundary partners, outcomes and activities (strategies) required to achieve its aim of transforming the workplace environment to prevent non communicable chronic diseases.

**Intentional design**

Vision

The wellness (health, work-life balance) of all employees (especially those at higher risk and their families) at the commercial plant is improved and sick days reduced. Employees are motivated and enabled to adopt a healthier lifestyle. The commercial plant is a trailblazer that innovates for the rest of the organization and other South African companies.

Mission

- The CIG will understand and help people to personalize their risk and to be motivated to consider lifestyle change for themselves and their families. The CIG will enable them to reduce their risky behaviors and risk factors for non-communicable diseases (NCDs).
- The CIG will focus on transforming the organizational environment to support healthier choices.
- The CIG will focus on low-cost, evidence-based, innovative, sustainable and accessible solutions that go beyond the current approaches
- The CIG will align their own lifestyle choices with the vision for the project.
- The CIG will continuously re-align itself with the vision and mission.

Boundary partners

- The onsite caterer and food supplier
- External support services
- Health and Wellness Department
- Management and decision makers

Outcome challenges

The outcome challenges are shown in Table 1.

**Table 1 Boundary partners and corresponding outcome challenges**

| **Outcome challenge 1: The onsite caterer and food supplier** |
| --- |
| The on-site caterers collaborate with Health & Wellness (H&W) to support the healthy lifestyle of employees by supplying fresh quality healthy meals daily. The caterers support the healthy lifestyles of employees by providing effective positive health messages/signage throughout all eating facilities. The caterer has a new menu with a variety of options included in the existing contract and is audited by the Heart Foundation. A dietician assists in developing the menu. The canteen has the look and feel of healthy eating that encourages a healthier lifestyle. The organization has a national food contract in place which advocates healthy living with improved subsidized meals. The caterers provide reasonably priced and flexible wellness food options at all canteens and kiosks. The food vendors on site offer a variety of healthy food options and are visible throughout the plant. Promoting healthy living is supported by sustainable planting and harvesting of employee vegetable patches on site. |
| **Outcome challenge 2: External support services** |
| All service providers have an awareness and understanding of the wellness strategy and actively support the drive. A list of the service providers is available, who share a similar vision and strategy in achieving healthy lifestyles. The commercial plant is a community and is influenced/supported/ developed by role players internal and external to the organization to achieve prevention and effective control of NCDs. |
| **Outcome challenge 3: Health and Wellness Department** |
| The H&W team are trained to communicate effectively with staff on healthy living. They collaborate strategically with stakeholders, such as management and information technology, in rolling out an effective media and communication strategy. The health and wellness communication strategy is innovative and focusses on preventative medicine. The team has identified roles and responsibilities, are easily accessible and visible during outages and are functional to the business operational outputs. The H&W team are role models for healthy lifestyles. Health and Wellness staff is fully trained in brief behavior change counselling, particularly in relation to NCDs. The medical referral system within the internal and external multidisciplinary team is structured and functional. The H&W team works well with the medical aids to link employee NCD risk profile to achieve effective prevention and control measures |
| **Outcome challenge 4: Management and decision makers** |
| Management has the health and wellness of their employees as an important part of their strategic vision and operational planning for the business. They understand that staff engagement and productivity are closely linked to their health and wellness, particularly NCDs such as diabetes, hypertension, ischaemic heart disease, cancer and mental illness. The managers themselves are role models for lifestyle change and promoting healthy behaviors. The managers are supportive of initiatives to reduce risky behaviors, risk factors and NCDs. There is effective engagement and collaboration with health and wellness. Health and wellness goals are included in the organizational business plan and personal development plans to ensure that they are prioritized and monitored. |

Progress markers

Progress markers were identified for all outcome challenges, and represented a change process for the four boundary partners, illustrating the progressive depth and complexity of changes in each of them from “expect to see”, to “like to see” and finally to what it would “love to see”. Changes that one would “expect to see” were more reactive to and predictable based on the direct actions of the CIG. Changes that one would “like to see” required some commitment to change from the boundary partner. Changes that one would “love to see” required a more sustained and transformative change in the boundary partner.

**Table 2 Progress markers for the boundary partners**

| **The onsite caterer and food supplier** |
| --- |
| **Expect to see** |
| 1. Collaborate with H&W Department to promote healthy eating and education. 2. Finance, Procurement, H&W to negotiate food contract with Fedics and their dietician 3. Benchmark with other like industries 4. Meet with facilities, environment, horticulture, safety for vegetable patches. 5. Collaborate with stakeholders for an innovative communication and education strategy |
| **Like to see** |
| 1. Approve and communicate the new Mission statement 2. Engage with stakeholders to include prevention and control of NCDs into the operational planning for the business plan. 3. Management provides support by providing time off, facility availability and upgrades. 4. Management actively supports the Health Risk Profiling by making personal lifestyle changes and promoting it to the organisation |
| **Love to see** |
| 1. The canteen has the look and feel of healthy eating with positive health messages/signage throughout all eating facilities 2. The organisation has a national food contract in place which advocates healthy living, audited by a dietician with improved subsidized meals. 3. Reasonably priced and flexible wellness food options at all canteens and kiosks. 4. The caterers offer a Wellness meal on Wednesday 5. The food vendors on site offer a variety of healthy food options and are visible throughout the plant (well wheels) 6. Promoting healthy living is supported by sustainable planting and harvesting of employee vegetable patches on site. |
| **External support services** |
| **Expect to see** |
| 1. Identify service providers aligned to the vision of preventing and controlling NCDs 2. Tap into existing groups and develop mutual understanding/agreements, similar to the agreement the Fire Team has with the City of Cape Town. 3. Medical practitioners, hospitals, local sports clubs, medical aids develop mutual agreements with KOU health & wellness team. 4. Collaborate with the local clinic 5. Establish process to initiate Parkrun at the commercial power plant. 6. Establish process to install an outdoor gymnasium 7. Identify areas with facilities management, nature conservation for routes for exercise, sport, walking, running and cycling routes 8. Request management support for extended lunch sport**,** participation and promotion of healthy lifestyle to prevent NCDs. 9. Management contributes the basic human and financial resources necessary to get the sporting infrastructure in place including the indoor gymnasium and portable showers. |
| **Like to see** |
| 1. Collaboration with stakeholder to roll out an effective communication strategy to promote sport and auxiliary services to assist employees to prevent and control NCDs 2. Innovative signage and marketing on the power plant for all activities and sport 3. Wellness Wednesday sports happening every week in conjunction with activities from H&W and Healthy food Wednesday. 4. Power plant Park run functioning and registered |
| **Love to see** |
| 1. Extended lunch every Wednesday (Wellness Wednesday), once a month sporting activity (cycling on the reserve, running, walking) 2. Vitality points for sporting activities 3. Designated gymnasium 4. Portable showers with identified trails for sporting codes. |
| **Health and Wellness Department** |
| **Expect to see** |
| 1. Establish the present state of the H&W center, benchmark with external wellness centers organizations business units and analyze current staff survey results 2. Consult with facilities and Wellness centre of expertise (COE) to determine the corporate ID that needs to be adhered to for transforming the look and feel of center, and establish resource availability 3. H&W staff attends training on brief behavior change counselling for prevention and control of NCDs. 4. Identify skills gaps with regard to communication skills, professionalism, roles and responsibilities. 5. Liaise with COE about IT service to incorporate technology using electronics to facilitate flow and running of H&W as a walk in center. |
| **Like to see** |
| 1. Allocated budget to be able to execute 2. H&W collaborate strategically with management and IT, in rolling out an effective, innovative media and communication strategy that focuses on prevention 3. H&W staff uses the behavior change tools in consultation. 4. Improve interpersonal contact and smooth process for screening and testing patients 5. Develop action plan according to survey results to engage with staff to include innovative preventative medicine strategies that are functional to the business operational outputs. 6. Consult with private medical insurers, e.g Vitality, Momentum, Bonitas to achieve effective prevention and control measures for NCDs |
| **Love to see** |
| 1. H&W collaborate strategically with management and IT, in rolling out an effective, innovative media and communication strategy that focuses on prevention 2. H&W staff uses the behavior change tools in consultation. 3. Improve interpersonal contact and smooth process for screening and testing patients 4. Develop action plan according to survey results to engage with staff to include innovative preventative medicine strategies that are functional to the business operational outputs. 5. Consult with private medical insurers, wellness partners, e.g. Vitality, Momentum, Bonitas to achieve effective prevention and control measures for NCDs 6. Have a vibrant internal environment with an inviting reception area, walk in center for quick check ups and external Zen garden 7. Have excellent customer service and role model healthy lifestyle. 8. H&W staff integrates key recommendations into their clinical practice 9. Visibility and effective engagement with staff on the plant and during outages 10. Incorporate technology into the practice. New electronic format of patient information and feedback |
| **Management and decision makers** |
| **Expect to see** |
| 1. Revise current strategic vision, and operational planning for the plant to include health and wellness. 2. Management knows their own health risk profiles 3. Management is educated on the impact of NCDs in the plant and how transformation will impact productivity and wellbeing of staff. 4. Management engage and collaborate with health and wellness regarding NCDs 5. Contributes adequate resources to the boundary partners to transform the workplace |
| **Like to see** |
| 1. Approve and communicate the OM mission statement. 2. Stakeholders to include prevention and control of NCDs into the operational planning for the business plan. 3. Management provides support by providing time off, facility availability and upgrades to support physical wellness. 4. Management actively supports the health risk assessment by making personal lifestyle changes and promoting it to the plant |
| **Love to see** |
| 1. H&W is included in the plant’s strategic vision and operational plan 2. Management are role models for lifestyle change, share experiences and benefits with rest of the organization. 3. Management support initiatives to reduce risky behaviors on NCD's 4. Include in the organizations key performance indicators to reward healthy lifestyle and behaviors. Look at the big organization plan of health and wellness and see how we are doing. |

Strategies

The initial strategies of the CIG that were planned in relation to each boundary partner are listed in Table 4. These strategies changed and evolved over time through the cycles of reflection and planning in the CIG.

**Table 3 Initial strategies for each boundary partner**

| **The onsite caterer and food supplier** |
| --- |
| 1. Collaborate with H&W to promote healthy eating 2. Collaborate with information technology and communications department to disseminate messages on health eating 3. Negotiate a revised contract with the catering company 4. Engage with catering manager and caterers to plan changes 5. Collaborate with employees to develop a vegetable garden |
| **External Support Services [medical practitioners, hospitals, sports clubs, nature reserve, businesses]** |
| 1. Collaborate with service providers (Western Cape on Wellness, local sports and running clubs) to provide active support to enhance physical wellness through sport. 2. Develop mutual agreements with the organization’s H&W team and private medical insurers 3. Collaborate with the onsite nature reserve for the inclusion of sport on the reserve. 4. Negotiate with management for an onsite gymnasium and portable exercise equipment |
| **Health and Wellness Department** |
| 1. Develop an effective media and communication strategy to promote H&W 2. Ensure that health risk assessments be mobile and accessible for all employees 3. Encourage H&W staff to be active role models for healthy lifestyles 4. Collaborate with private medical insurers on NCD prevention programs 5. Negotiate with H&W staff to attend brief behavior change counselling training to assist employees in reducing NCDs |
| **Management and decision makers** |
| 1. Negotiate for H&W to be prioritised as a standing item in meetings. 2. Negotiate for the inclusion of H&W activities in meetings, work team sessions and capacity building program. 3. Synchronise the H&W activities with the organisations calendar 4. Present health risk assessments findings and implications to the executive committee 5. Personalise the health risks for managers and motivate them personally. 6. Negotiate for outage schedules, rosters and incentives to be aligned with H&W program. |

Organizational practices

In this context organizational practices referred to the functioning of the CIG. The CIG aligned itself with eight criteria for its own practice as shown in Table 4.

**Table 4: Quality criteria for the functioning of the CIG**

| **Quality criteria** | **Definition** |
| --- | --- |
| Alignment with purpose | Alignment of the group members with the purpose of the research both drives the process and acts as the contract between the members. Aligning oneself with a particular outcome or personal intention, rather than the purpose of the research, may lead to a lack of openness in the inquiry. |
| Ownership of the inquiry process | Ownership of the research by members of the group is crucial to the quality of the inquiry. The initiating researcher will need to transfer power, knowledge of the research methodology, ownership of the research questions and process so that after the group is established he or she does not dominate the inquiry. |
| Development of reflectivity | As the members of the group are both the researchers and the researched, the quality of the inquiry will depend on their ability to witness themselves. This requires a reflective stance that is characterised by heightened awareness, open-mindedness, critical questioning and commitment to dialogue. |
| Democratic and collaborative group dynamics and facilitation | The facilitator must strive for a genuine collaborative and democratic group process. The level of trust will be related to telling the truth without judgement and staying within the common purpose. Breaking this contract with each other leads to a loss of trust and commitment and the facilitator must guard against this. |
| Commitment to practical action and experience | The group must be committed to a balance of both action and reflection. Some groups may find it easy to take action, but difficult to pause for adequate documentation and reflection. Others may be good at planning and reflecting, but short on actually engaging with the practical action. |
| Documentation of the process | The following 3 aspects must be documented: The individual experience and action. The group process and dynamics. The developing reflections, research statements and conclusions. The quality of the research process is made publicly accountable through documentation. |
| Transferability | Transferability is another aspect of quality whereby the group’s findings should be sufficiently clearly described to enable readers to understand what aspects of the inquiry can be appropriated to their own context. The reporting of this research therefore should be in its “rich contextual detail”. |
| Construction of practical knowledge | The purpose of co-operative inquiry is to construct practical knowledge through cycles of action and reflection. Therefore one way of judging the quality of the research is in the practical usefulness of this new knowledge. The way in which this consensus was constructed will also reflect on the quality of the inquiry. |

Source: Mash B. African primary care research: participatory action research. Afr J Prm Health Care Fam Med. 2014;6(1), Art. #585, 5 pages. http://dx.doi.org/10.4102/ phcfm.v6i1.585
